# Study on the protective effect and mechanism of Liriodendrin on radiation enteritis in mice

**DOI:** 10.1093/jrr/rrab128

**Published:** 2022-01-21

**Authors:** Jiajun Li, Xin Zheng, Xiong Li, Jing Yang, Wei Liu, Lei Yang, Bin Liu

**Affiliations:** Department of General Surgery, The Second Affiliated Hospital of Chengdu Medical College, China National Nuclear Corporation 416 Hospital, Chengdu, Sichuan, 610051, China; Department of General Surgery, The Second Affiliated Hospital of Chengdu Medical College, China National Nuclear Corporation 416 Hospital, Chengdu, Sichuan, 610051, China; Department of General Surgery, The Second Affiliated Hospital of Chengdu Medical College, China National Nuclear Corporation 416 Hospital, Chengdu, Sichuan, 610051, China; The School of Biological Science and Technology, Chengdu Medical College, 610083, Chengdu, China; Department of General Surgery, The Second Affiliated Hospital of Chengdu Medical College, China National Nuclear Corporation 416 Hospital, Chengdu, Sichuan, 610051, China; Tianjin key Laboratory of Acute Abdomen Disease Associated Organ Injury and ITCWM Repair, Institute of Acute Abdominal Diseases, Tianjin Nankai Hospital, Tianjin, 300100, China; Department of General Surgery, The Second Affiliated Hospital of Chengdu Medical College, China National Nuclear Corporation 416 Hospital, Chengdu, Sichuan, 610051, China

**Keywords:** Liriodendrin, radiation enteritis, anti-apoptosis, anti-inflammatory activity, sphingolipid

## Abstract

Patients receiving pelvic or abdominal radiotherapy may experience acute and/or chronic side effects due to gastrointestinal changes. However, effective medicine for treating radiation enteritis has not been found yet. Sargentodoxa cuneata is a famous Chinese medicine used to treat intestinal inflammation, and our research team has found the main biologically active compound through its extraction, which is Liriodendrin. In this study, we found that Liriodendrin can reduce the expression of Cer, Cer1P and S1P in the sphingolipid pathway, thereby reducing the histological damage to the intestinal tract of mice and inhibiting the apoptosis of intestinal tissue cells. In addition, Liriodendrin can reduce the levels of pro-inflammatory cytokines (IL-6 and TNF-α), and it is suggested through flow cytometry that the proportion of neutrophils in the intestinal tissue can decrease due to the existence of Liriodendrin. At the same time, the western blot evaluation revealed that Liriodendrin significantly inhibited the activation of Bcl-2/Bax/Caspase-3 and NF-κB signaling pathways. The results show that Liriodendrin can inhibit intestinal inflammation and intestinal cell apoptosis through the sphingolipid pathway. Therefore, the aforementioned results demonstrated that Liriodendrin may be a promising drug for the treatment of radiation enteritis.

## INTRODUCTION

As one of the main damage sites during radiotherapy, the small intestine is very sensitive to radiation. Ionizing radiation damage to the gastrointestinal tract is a common side effect of radiotherapy, and a considerable proportion of patients suffer from acute or chronic gastrointestinal symptoms [1]. Acute radiation enteritis is mainly caused by repeated damage to the intestinal mucosa, which is associated with ionizing radiation and its complicated healing mechanism [2]. However, there is no widely recognized drug with a better effect on the treatment of radiation enteritis at present. Therefore, the purpose of studying the inflammatory mechanism of radiation enteritis is to improve the effectiveness of radiation therapy and to prevent the occurrence of radiation enteritis.

Another important feature of radiation enteritis is the cytokine-mediated process. The inflammatory response is characterized by the influx of white blood cells from the inflammation site. When the local cells are damaged, the cascade of inflammation causes the secretion of cytokines. These cytokines increase the adhesive molecules on the blood vessel surface and promote the entry of white blood cells into the damaged tissue [3]. Radiation activates NF-κB, ERK/MAPK, PI3K/AKT, SAPK/JNK and other signaling pathways by interacting with intestinal epithelial cells, leading to the transcription and expression of many cytokines such as pro-inflammatory factors, growth factors, chemokines and apoptosis factors, thus the balance between pro-inflammatory factors and anti-inflammatory factors is broken. During radiotherapy, the patients’ small intestinal epithelial cells will experience degeneration, necrosis and apoptosis, and then the function of intestinal wall barrier will be lost, and even death due to bacteremia and septicemia [4–6]. Studies have found that NF-κB, AP-1 and SOCS3 are activated during radiotherapy. After 6 hours, high expression of IL-6 and TNF-α can be detected in the intestinal mucosa, and high levels of IL-6 and TNF-α can be sustained for 3 days [7]. Apoptosis is mainly caused by the overexpression of BAX and the decrease of Bcl-2, which plays an important role in acute radiation enteritis [8].

Ceramide is a simple membrane sphingolipid that constitutes the backbone of all complex sphingolipids [9]. High levels of ceramide can trigger programmed cell death [10]. Ceramidase hydrolyzes ceramide to produce free fatty acids and sphingosine, which are then phosphorylated by sphingosine kinase (Sphk) to produce sphingosine-1-phosphate (S1P), a kind of survival with intracellular and extracellular functions Lipid mediators have the effect of promoting inflammation [11, 12]. Ceramide is acted on by ceramide kinase to generate ceramide-1-phosphate (Cer1P), and Cer1P has the effect of promoting the development of inflammation. Endothelial cell apoptosis in radiation enteritis depends on the sphingolipid ceramide produced by acid sphingomyelinase (ASM) [13]. Within a few minutes after exposure to radiation, the pool of ASM initially contained in the lysosome transfers on the outer plasma membrane that hydrolyzes sphingomyelin to ceramide. Once produced, ceramide can activate p38 MAPK-dependent apoptosis [14, 15]. When an anti-ceramide monoclonal antibody is administered *in vivo*, the formation of a ceramide-rich membrane platform is prevented, and similar effects can be observed *in vivo* [16]. In summary, these studies clearly highlight the critical role of ceramide in radiation-induced gastrointestinal toxicity. However, the precise mechanism regarding ceramide-induced apoptosis or inflammation of intestinal epithelial cells still remains unclear.

Traditional Chinese medicine is a valuable asset of Chinese medicine. Many traditional Chinese medicines or natural products have anti-inflammatory effects. The extraction of Chinese medicine monomers from plants might be promising candidates to develop new drugs for the treatment of radiation enteritis. In our previous study, Liriodendrin was extracted from Sargentodoxa cuneata as is one of the active ingredients [17]. Liriodendrin has a variety of biological functions, including anti-inflammatory, anti-oxidant, anti-tumor, anti-fungal, anti-platelet aggregation and even anti-Alzheimer’s effect [18–21]. The former study of our research group suggested that Liriodendrin can reduce the content of VEGF and NF-κB in mouse lung tissues, suppress the expression of pro-inflammatory factors and significantly improve the survival rate of septic mice induced by cecal ligation and puncture. Further studies have shown that Liriodendrin can down-regulate NF-κB signaling in lung macrophages stimulated by LPS (Lipopolysaccharide), and reduce the production of pro-inflammatory mediators [22]. Our previous research also proved that Liriodendrin can prevent and improve the protective effect of dextran sulfate (DSS)-induced ulcerative colitis in mice [23]. However, the effect of Liriodendrin on radiation enteritis has not been reported yet.

In this study, the protective effect of Liriodendrin on radiation enteritis was firstly explored, which is through the sphingolipid pathway. Its inhibition of intestinal apoptosis and inflammation was also revealed by inhibiting the Bcl-2/Bax/Caspase-3 and NF-κB signaling pathways. Those mechanisms show prospects for applying traditional Chinese medicine in treating radiation enteritis.

## MATERIALS AND METHODS

### Drug and antibody

As mentioned earlier, Liriodendrin was extracted from Sargentodoxa cuneata and dissolved in Dimethyl sulfoxide (DMSO) [17]. The purity of liriodendrin was determined to be over 97% by normalization of the peak areas by HPLC and its structure was elucidated by the NMR. All western blot antibodies were purchased from Cell Signaling technology (USA). The BCA protein detection kit was provided by Thermo Scientific (USA).

### Animals and radiation

C57BL/6 J mice were purchased from CHENGDU DOSSY EXPERIMENTAL ANIMALS CO., LTD., China. Female mice are 6- to 8-weeks-old, weighing about 20 ± 3 g. Notably mice were under SPF management, and adapted to the environment in a specific pathogen-free cage for at least 1 week before the experiment. Eighteen mice were randomly divided into three treatment groups: control group, IR group, IR + LD group (100 mg/kg). Mice in the IR + LD group were orally administered Liriodendrin (100 mg/kg) 3 days before irradiation, and the following 7 days after irradiation. The experiment was conducted in accordance with the rules and regulations established by the Medical Ethics Committee of the Second Affiliated Hospital of Chengdu Medical College.

The irradiation was performed using a co-60 source placed in the GWXJ80 co-60 teletherapy machine (Nuclear Power Institute of China) in the Second Affiliated Hospital of Chengdu Medical College, with an output dose rate of 39.22Gy per minute and irradiation dose of 8Gy. The treatment of mice that received fake radiation was similar to that of mice that received radiation except radiation. Afterwards, the mice were returned to the animal room for daily observation and treatment.

### Histological analysis

After the mice were sacrificed, the small intestine tissues were fixed with 10% formalin and embedded in paraffin. The sections were stained with hematoxylin and eosin (H&E) and analyzed under an optical microscope. According to the previous description [22], for morphological analysis, each mouse blindly analyzed six circular cross-sections from the encoded H&E-stained digital photos to measure the number of crypts using ImageJ 1.37 software.

### Sphingolipid group analysis of mice intestinal tissue (Cer, Cer1P, S1P)

The collected ileum tissues were cryopreserved in liquid nitrogen, and five samples from each group were randomly selected, then 15 samples in three groups were collected under dry ice storage and sent to Changzhou Zhongke Zhidian Biotechnology Co., Ltd., China for sphingolipid group analysis. Lipids from the samples were extracted with an improved Bligh/Dyer extraction method (two extractions). The samples were reconstituted in the isotope mixture standard, and all analyses were performed in the Electrospray Ionization (ESI) mode using an Exion UPLC-QTRAP 6500 Plus (Sciex) LC/MS instrument [24]. Phenomenex Luna silica 3 μm (inner diameter 150×2.0 mm) chromatographic column was applied to separate various polar lipids under different conditions. Mass spectrometry multiple reaction monitoring (multiple IRactionmonitoring [MRM]) was established for the identification and quantitative analysis of various lipids [25, 26]. The quantification of lipid substances is carried out by the added internal standard.

**Fig. 1.**
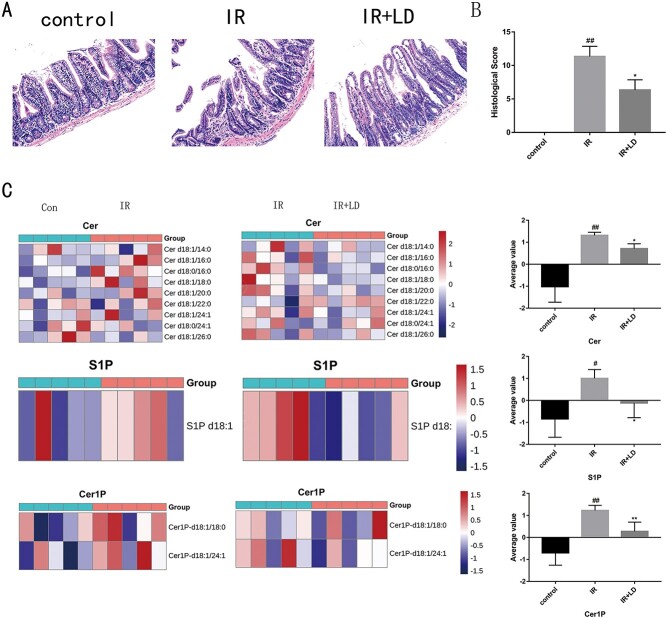
The protective effect of Liriodendrin on radiation enteritis in mice (100 μm). (A) the effect of Liriodendrin on the pathological damage of the intestinal tract of mice; (B) the histological score of pathological damage. (##: IR group vs control group *P* < 0.01; ^*^: IR + LD group vs IR group *P* < 0.05). (C) Compared with the control group, the expressions of Cer, Cer1P and S1P were increased in the IR group. Compared with the IR group, the IR + LD group found that Liriodendrin can reduce the expression of Cer, Cer1P and S1P. (##: IR group vs control group *P* < 0.01; #: IR group vs control group *P* < 0.05; ^**^: IR + LD group vs IR group *P* < 0.01; ^*^: IR + LD group vs IR group *P* < 0.05).

### Western blots

Protein was extracted from the small intestine incubated by adding cold RIPA lysis buffer (Solarbio Science and Technology, Beijing, China) containing protease and phosphatase inhibitors. The total protein concentration was determined with BCA kit (Thermo Scientific, USA). According to the calculated protein concentration, samples containing the same amount of protein (20 μg) were mixed with the loading buffer for gel electrophoresis, and the protein was transferred to a PVDF membrane (Millipore, USA). These membranes were blocked with 5% milk and 0.1% Tween 20 in Tris buffer, and then with the primary antibody at 4°C, namely anti-NF-κB, anti-p-NF-κB, anti-BAX, anti-Bcl-2, anti- Caspase3 (1:1000 dilution, CST, USA) and anti-GADPH (1:10000 dilution, CST, USA) and were incubated overnight. Then appropriate horseradish peroxide-conjugated secondary antibody was added at room temperature. Finally, chemiluminescent substrates were used to detect proteins (Bio-Rad, USA). The gray value was analyzed using Quantity One software (Bio-Rad, USA).

### Real-time quantitative reverse transcription

Total RNA was extracted from the small intestine tissue using TRIzol RNA reagent (Takara-bio, Japan) according to the instructions. Reverse transcription kit (Thermo Scientific, USA) was used to reverse transcription of mRNA into cDNA accordingly. Real-time PCR was detected by ABI 7500 fast Real-time PCR system (Thermo Scientific, USA). Primers were synthesized as follows: IL-6 (F) 5′-AGTTGCCTTCTTGGGACTGA-3′ and (R) 5′-TCCACGATTTCCCAGAGAAC-3′; TNF-α (F) 5′- CGTCAGCCGATTTGCTATCT-3′ and (R) 5′-CGGACTCCGCAAAGTCTAAG-3′. The expression of mRNA was quantified by 2 − ΔΔCt.

### Flow cytometry

The small intestine tissue was digested with collagenase to prepare a single cell suspension, and the NovoCyte flow cytometer was used for detection (ACEA Biosciences, USA). The surface of neutrophils was labeled with monoclonal antibodies (Sungene Biotech, China). The label protocol is as follows: CD11b-FITC, Ly6G-PE and CD45-APC.

### TUNEL assay

The pathological tissue sections (3 μm thick) were processed using the manufacturer’s method (Solarbio Science and Technology, Beijing, China). Finally, the sections were analyzed with an optical microscope.

### Statistical analysis

All data have at least three independent experiments, and all results are expressed as mean ± SEM. The data was analyzed by SPSS 19.0 software and Graph Pad Prism 5.0. One-way analysis of variance (ANOVA) was used between multiple groups, and Tukey’s multiple comparisons test was used between the two groups. *P* < 0.05 was considered statistically significant.

## RESULTS

### The protective effect of Liriodendrin on radiation enteritis in mice

The severity of radiation enteritis was evaluated by analyzing H&E staining. In addition to principal component analysis, heat maps are often used in omics studies to observe the overall differences of samples, which facilitates the discovery of outliers. Heat maps show metabolites in two colors. Since the content of different metabolites varies greatly, all metabolites are firstly standardized so that the average value of metabolite in all samples will be white if it is close to 0. It would be negative if metabolite is lower than the average value and otherwise positive. The darker the color is, the bigger the difference from the average value is. The color bar on the right of the heat map indicates the corresponding value of the color. The meaning of the value indicates the number of standard deviations from the average value. Our Research has found that Cer, Cer1P and S1P have different changes. As shown in Fig. 1A, no histopathological damage was observed in the control group. The IR group induced serious damage to the small intestine tissues, such as damage to the intestinal epithelium, reduced number of crypts and villi loss. However, Liriodendrin attenuates the pathological damage of the small intestine. Fig. 1B shows the histological statistical scores of pathological damage. Compared with the IR group, the treatment of Liriodendrin can effectively attenuate the pathological damage caused by radiation (*P* < 0.01). As suggested in Fig. 1C, Liriodendrin can protect the intestinal tract by regulating sphingolipids, and it is found that Cer, Cer1P and S1P have different changes. That is, the content of Cer, Cer1P and S1P in the IR group is higher than the average, while the content of Cer, Cer1P and S1P in the control group is lower than the average. After treatment with Liriodendron, it suggested that the content of Cer, Cer1P and S1P in IR + LD group decreased.

### Liriodendrin inhibits the expression of phosphorylated NF-κB and the expression of pro-inflammatory cytokines in the small intestine tissue

**Fig. 2.**
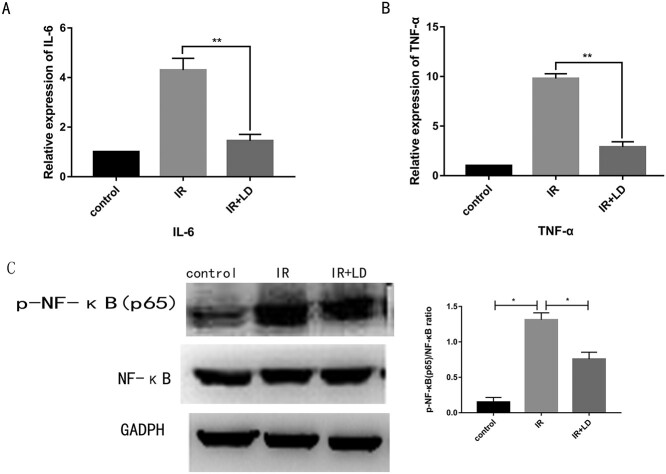
During radiation enteritis, Liriodendrin can inhibit the production of p-NF-protein and pro-inflammatory cytokines. (A) Liriodendrin reduces the content of p-NF-κB protein. (B, C) Liriodendrin reduces the mRNA levels of the pro-inflammatory cytokines IL-6 and TNF-α. The western blotting experiment and real-time PCR detection were repeated (^*^P < 0.05, ^**^P < 0.01).

In order to further explore the mechanism of Liriodendrin against the inflammation of small intestine tissue, we examined the amount of NF-κB and p-NF-κB protein by western blotting. Real-time-PCR technology was used to detect the mRNA content of major pro-inflammatory cytokines. As shown in Fig. 2A–C, after the mice were irradiated for 3 days, the amount of p-NF-κB protein increased, and the mRNA content of IL-6 and TNF-α increased significantly. However, after treatment with Liriodendrin, the content of p-NF-κB protein and pro-inflammatory cytokines was significantly reduced (^*^P < 0.05, ^**^P < 0.01).

### Liriodendrin reduces the infiltration of neutrophils in mice with radiation enteritis

**Fig. 3.**
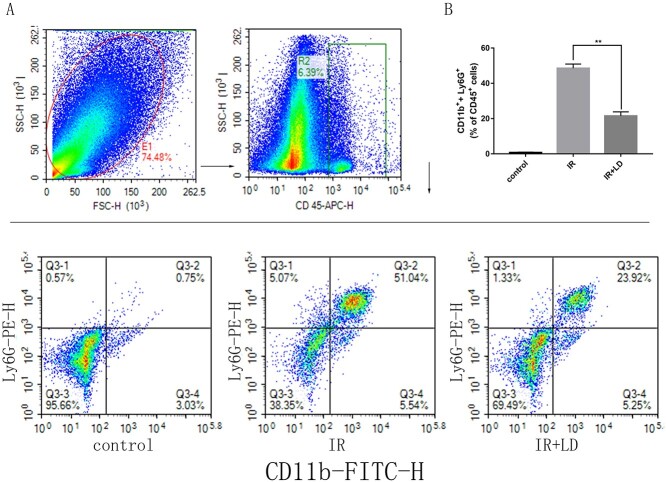
Liriodendrin reduces the proportion of neutrophils in the small intestine tissue. CD45+/CD11b+/Ly6G+ cells represent neutrophils. (A) The proportion of neutrophils in the small intestine tissue of each group. (B) Comparison of the proportion of CD11b+ + Ly6G+ cell population to CD45+ cell population in small intestine tissue (^**^P < 0.01).

Neutrophils play an important role in inflammation. We analyzed the number of neutrophils in small intestine tissue by flow cytometry to further evaluate the severity of radiation enteritis. As shown in Fig. 3A, we found that compared with the IR group, the neutrophils number in the small intestine tissue of Liriodendrin treated mice was reduced, which means the number of CD11b^+^ and Ly6G^+^ cells in CD45^+^ cells declined.

### Liriodendrin reduces apoptosis in the small intestine

**Fig. 4.**
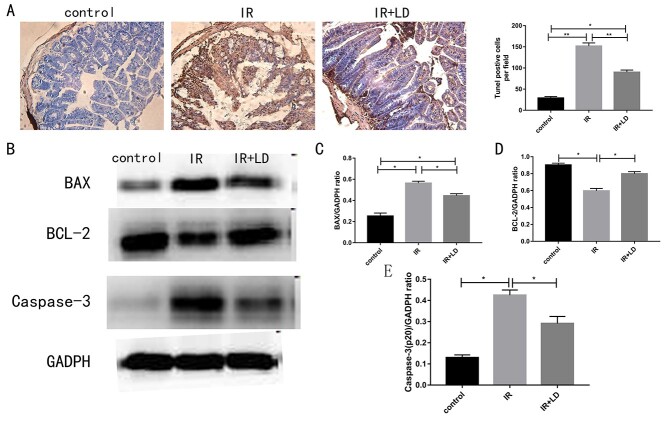
Liriodendrin can reduce cell apoptosis after radiation. (A) Liriodendrin reduces radiation-induced apoptosis (100 μm). The apoptosis was analyzed by the TUNEL assay and the number of TUNEL+ cells was counted. The results are drawn from at least 15 areas. (B) Liriodrin can down-regulate the levels of BAX and Caspase-3, and up-regulate the levels of Bcl-2. Quantification of BAX (C), BCL-2 (D) and Caspase-3 (E). (^*^ < 0.05, ^**^  *P* <0.01).

To further validate our observations, we evaluated cell apoptosis in the small intestine by TUNEL analysis. In order to explore the mechanism of Liriodendrin in inhibiting small intestinal cell apoptosis, we analyzed the expression of related proteins in the Bax/Bcl-2/Caspase-3 pathway by Western blot. As shown in Fig. 4A, Liriodendrin can prevent radiation-induced intestinal damage by inhibiting cell apoptosis. Fig. 4B–E suggested the results of immunoblotting experiments. It could be seen that Liriodendrin can reduce the levels of BAX and Caspase-3 and increase the levels of Bcl-2 (*P* < 0.05).

## DISCUSSION

Patients underwent pelvic or abdominal radiotherapy may encounter acute and/or chronic side effects due to gastrointestinal changes. Ionizing radiation damage to the gastrointestinal tract is a common side effect of radiotherapy, and consequently a significant proportion of patients suffer from acute or chronic gastrointestinal symptoms. However, there is no widely effective drug for the treatment of radiation enteritis. In this study, Liriodendrin reduced the intestinal damage caused by radiation. These effects may lie on the inhibition of inflammatory factors and inhibition of cell apoptosis.

Under physiological conditions, Liriodendrin can promote the restoration of the structure of the small intestine of mice. Radiation enteritis is essentially intestinal inflammation induced by external radiation damage and cytokine-mediated. Intestinal tissue exposed to radiation will produce reactive ions, which combine with water molecules in cells to form free radicals such as hydroxyl groups. These free radicals are believed to be responsible for DNA breakage and cell death. Secondary to radiation exposure, the gene responsible for the translation of transforming growth factor (TGF-β) is activated. This activation stimulates collagen and fibronectin genes to promote fibrosis [27]. The most characteristic pathological changes of radiation enteritis are intestinal epithelial fibrosis and endarteritis obliterans [8]. The initial changes can be observed 2–3 hours after irradiation, mainly to inhibit the apoptosis of crypt epithelial cells. As time goes by, apoptotic fragments composed of nucleus or cytoplasmic fragments and cytoplasmic condensation can be seen [8, 28, 29]. High levels of ceramide can trigger programmed cell death [10]. The apoptosis of endothelial cells in radiation enteritis depends on the sphingolipid ceramide produced by ASM [13]. Extracellular sources of ASM and ceramide can produce harmful reactions to target cells [15]. The smpd1 gene not only produces lysosomal ASM (L-ASM), but also produces secretory ASM (S-ASM) through differential transport of common protein precursors. S-ASM is mainly produced by endothelial cells, and its secretion is up-regulated under inflammatory conditions [30, 31]. In contrast, extracellular L-ASM is the result of non-specific release from damaged cells [32]. *In vivo*, ASM subtypes and ceramides have been detected in most body fluids [33]. In this study, the detection of the sphingolipid pathway in the intestinal tract of mice with radiation enteritis suggested changes in Cer, Cer1P and S1P. It was found that Liriodendrin can inhibit the expression of Cer, Cer1P and S1P, suggesting that Liriodendrin may be anti-inflammatory and anti-apoptosis to play a role. At the same time, we found that the release of pro-inflammatory factors IL-6 and TNF-α was inhibited after treatment with Liriodendrin. In addition, TUNEL experiments show that Liriodendrin can inhibit small intestinal cell apoptosis and prevent intestinal radiation damage.

Radiation therapy can also cause the recruitment of macrophages, CD3+ T cells, CD34+ hematopoietic progenitor cells and the infiltration of neutrophils in the intestinal epithelial mucosa, and it can also promote inflammation and vascular damage [34]. Neutrophils play an important role in inflammation. The activated macrophages produce antimicrobial molecules and release chemokines and cytokines to enhance the activated recruitment of neutrophils. Antimicrobial molecules and recruited neutrophils can kill pathogens and eliminate infections and dead cells. It was proved by flow cytometry that the Liriodendrin group can reduce the proportion of centrioles in the small intestine tissue to protect the intestinal from radiation damage.

According to current mechanism, radiation generates oxygen free radical ROS through the interaction with water molecules in tissue cells. ROS produced by low concentration or transient acts as the second messenger, activating NF- B, ERK/MAPK, PI3K/AKT, BAX/Bcl-2/Caspase-3 and other signaling pathways. The changes in these signaling pathways cause the transcription and expression of many cytokines such as pro-inflammatory factors, growth factors, chemokines and apoptosis factors, leading to a cascade of mucosal inflammation [7]. In this study, Liriodendrin treatment can suppress the expression of BAX and Caspase-3, promote the expression of BCL-2, and inhibit the phosphorylation of NF-κB. It is suggested that Liriodendrin can regulate the caspase apoptosis pathway induced by radiation.

In summary, Liriodendrin can reduce the severity of radiation enteritis by reducing the damage of mouse small intestine tissue, inhibiting cell apoptosis and anti-inflammatory effects through the sphingolipid pathway. Therefore, Liriodendrin is a promising drug for the treatment of radiation enteritis.
